# Kappa free light chains in cerebrospinal fluid to detect inflammation - results from a multicentric prospective real-world study

**DOI:** 10.3389/fimmu.2026.1806398

**Published:** 2026-03-18

**Authors:** Marie Süße, Malte Johannes Hannich, Felix Lottermoser, Stefan Gross, Kathrin Budde, Irena Crnkovic-Mertens, Margot Thiaucourt, Markus Roser, Franz Felix Konen, Thomas Skripuletz, Rayan Suliman, Peter Mirtschink, Henry Bock, Karl Reuner, David Poitz, Tjalf Ziemssen, Hayrettin Tumani, Jan Lewerenz, Michael Khalil, Andrea Harrer, Matthias Nauck, Alexander Dressel

**Affiliations:** 1 Department of Neurology, University Medicine Greifswald, Greifswald, Germany; 2 Institute of Clinical Chemistry and Laboratory Medicine, University Medicine Greifswald, Greifswald, Germany; 3 Department of Internal Medicine B, University Medicine Greifswald, Greifswald, Germany; 4 German Center for Cardiovascular Research (DZHK), Partner Site Greifswald, Greifswald, Germany; 5 Medical Care Center, Laboratory Dr. Limbach and Colleagues GbR, Heidelberg, Germany; 6 Institute of Laboratory Medicine, SLK-Kliniken Heilbronn, Heilbronn, Germany; 7 Department of Neurology, Medical School Hannover, Hannover, Germany; 8 Institute of Laboratory Medicine, University Hospital Dresden, Dresden, Germany; 9 Institute of Laboratory Medicine, Medical University Lausitz- Carl Thiem, Cottbus, Germany; 10 Centre of Clinical Neuroscience, Department of Neurology, Medical Faculty and University Hospital Carl Gustav Carus, Technical University of Dresden, Dresden, Germany; 11 Department of Neurology, University of Ulm, Ulm, Germany; 12 Department of Neurology, Medical University of Graz, Graz, Austria; 13 Department of Neurology, Christian-Doppler University Hospital, Paracelsus Medical University, Centre for Cognitive Neuroscience, Salzburg, Austria; 14 Department of Dermatology and Allergology, Paracelsus Medical University, Salzburg, Austria; 15 Department of Neurology, Medical University Lausitz- Carl Thiem, Cottbus, Germany

**Keywords:** cerebrospinal fluid, free light chain kappa, intrathecal immunoglobulin synthesis, oligoclonal IgG, FLCk synthesis

## Abstract

**Background:**

Free light chain kappa (FLCκ) have emerged as a sensitive biomarker to detect intrathecal immunoglobulin synthesis. To evaluate the role of FLCκ in routine cerebrospinal fluid laboratory diagnostics we conducted a prospective nationwide real world diagnostic study for evaluating the integration of kappa free light chain into clinical routine (ORCAS - prospective multicenter validation of a new laboratory workflow integrating the free light **c**hains k**a**ppa in CSF analysis).

**Objectives:**

To determine whether testing for intrathecal synthesis of FLCκ in cerebrospinal fluid (CSF) can predict intrathecal immunoglobulin (Ig) synthesis of total IgG, IgA, IgM or CSF-specific oligoclonal bands (OCB) with high sensitivity.

**Materials & Methods:**

FLCκ were measured in 1052 paired CSF and serum samples according to local laboratory standards in six laboratories in Germany. Sensitivity and negative predictive value (NPV) of intrathecal FLCκ synthesis as first line detection of intrathecal Ig synthesis were assessed.

**Results:**

Of the 1052 samples, 624 fulfilled the inclusion criteria. The intrathecal fraction of FLCκ predicted intrathecal Ig synthesis with a sensitivity of 0.87 (CI 0.81-0.93) and a NPV of 0.97 (CI 0.95-0.98). The sensitivity for predicting CSF-specific OCB was 0.93 (CI 0.88-0.98).

**Conclusions:**

In the real world setting this study analyzing FLCk for detecting intrathecal Ig synthesis did not reach its prespecified primary endpoint of sensitivity >0.95. Therefore, FLCκ should not yet be introduced as a stand-alone preselection marker for intrathecal Ig synthesis, but may provide additional value when combined with established diagnostic parameters.

## Introduction

The proof of an intrathecal immunoglobulin (Ig) synthesis is an indicator for central nervous system (CNS) inflammation. In current laboratory routine, Ig synthesis is assessed using the Ig quotient diagrams or the determination of oligoclonal bands (OCB) (1, 2). Recently, free light chain kappa (FLCκ) has been introduced as an additional biomarker for intrathecal Ig synthesis (3) and a hyperbolic FLCκ quotient diagram was developed to identify pathological intrathecal fractions (IF) (4). We previously demonstrated that the FLCκ IF predicts intrathecal Ig synthesis with high sensitivity of 98% and absence of Ig synthesis with a probability of 99.5% (5). We developed a workflow in which determining FLCκ IF serves as initial step and criterion whether cerebrospinal fluid (CSF)/serum sample pairs require additional analysis with Ig and OCB analysis (5).

In this paper, we report the primary endpoint of a multicenter prospective biomarker study (ORCAS study - prospective multicenter validation of a new laboratory w**or**kflow integrating the free light chains kappa in CSF analysis) (6).

## Materials and methods

### Standard protocol approvals and patient consent

Data obtained from CSF and serum samples collected in 6 German laboratories participating in the ORCAS study were included. This study was performed in accordance to the statements of the local ethics research committees (**Supplementary Table S1**). The study design was based on the STARD criteria (STAndards for Reporting of Diagnostic Accuracy studies) (7). A preprint of the study protocol was posted on July 17, 2023 on the medRxiv preprint server (6).

### Data collection

This study included consecutive CSF and serum samples arriving for routine diagnostic purposes between July 2023 and March 2024 irrespective of the clinical diagnostic question. Since the primary endpoint was the detection of Ig synthesis using FLCκ in all obtained sample pairs, no subanalyses were performed on the performance of FLCκ as a biomarker in specific neuroinflammatory diseases. All laboratory diagnostic tests including albumin, Ig and FLCκ in serum and CSF to calculate intrathecal synthesis and OCB analysis were performed in each laboratory according to local standards (**Supplementary Table S1**). The data was anonymized before being transmitted to the main study site (University Medicine Greifswald).

**Figure 1:**
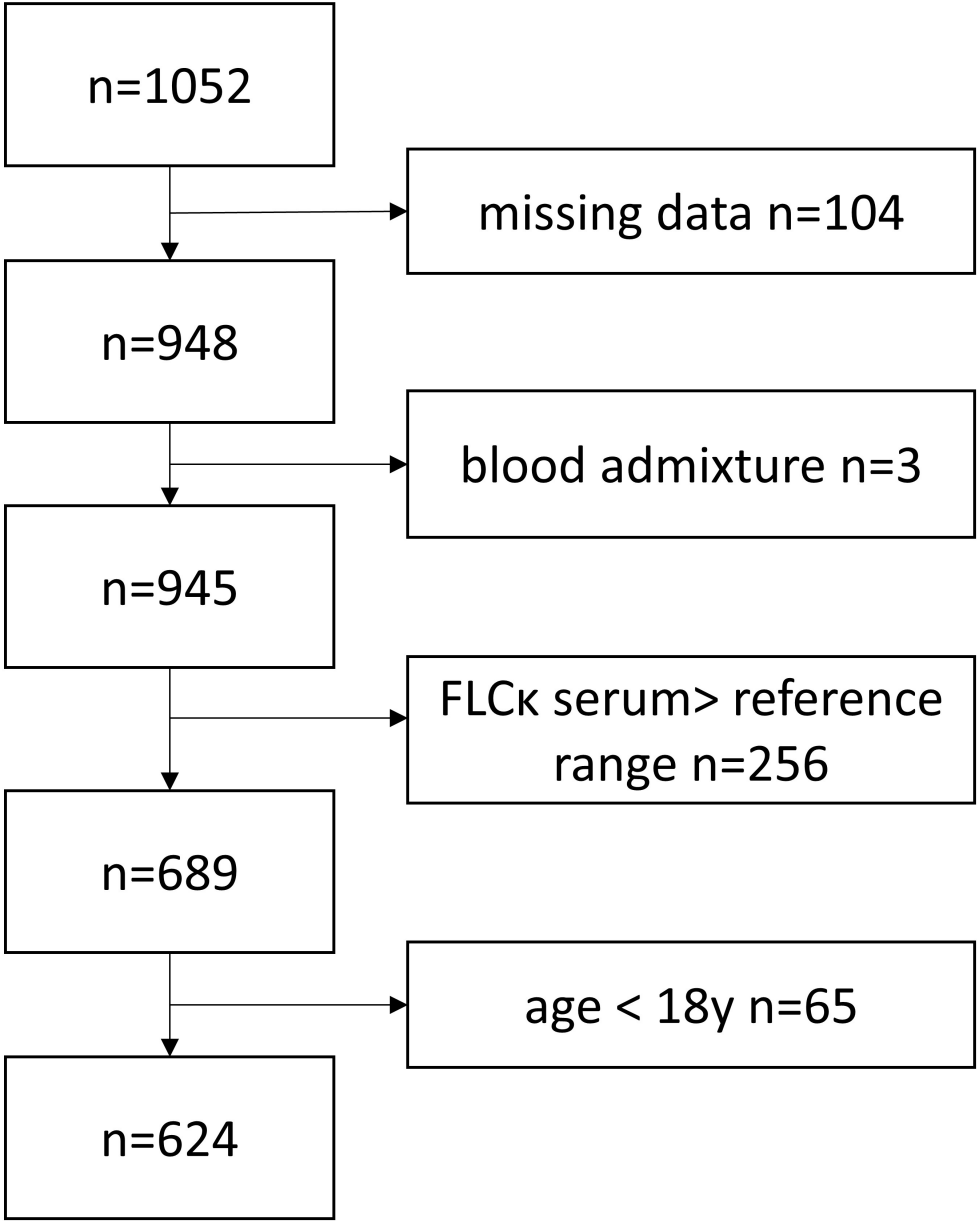
Flow chart illustrating the inclusion and exclusion of study participants.

In total, data for 1052 CSF/serum sample pairs were transmitted. Of those, 169 data sets were excluded because data sets were incomplete or did not meet demographic inclusion criteria (age >18 years). An additional 256 data sets were excluded due to serum FLCκ above the reference range (**Supplementary Table S1**), since an inclusion entails the risk of reducing the sensitivity (8). Three data sets were excluded due to blood contamination (**Figure 1**). CSF FLCκ values below the lower limit of detection (LLOD) (n=79/1052; 7.5%) were replaced with the value of the LLOD (**Supplementary Table S1**, **S2**).

### Laboratory analyses

The reference range of FLCκ and the FLCκ IF was calculated using the established FLCκ quotient diagram (4). The presence of an intrathecal immune response was defined as either presence of CSF-restricted OCB according to local standards or an intrathecal fraction of IgG, IgM, IgA in the respective quotient diagrams.

### Statistical analysis

Consecutive testing is expected to result in a low prevalence of an intrathecal humoral immune response. Therefore, both the negative predictive value (NPV) and sensitivity were defined as co-primary endpoint (6).

A calculation of the minimum required sample size was based on the precision of two-sided confidence intervals (CI). Since sensitivity and NPV were defined as co-primary endpoints (6), two-sided 97.5% CIs were applied (global α ≤ 0.05). The desired precision was defined as a maximum CI width of 0.05. Based on an assumed sensitivity and NPV of 95%, a sample size of 424 was calculated to yield a two-sided 97.5% CI with a width of 0.05 (0.921–0.971) for each parameter.

RStudio (R version 3.5.1 2018-07-02) and IBM SPSS Statistics version 29 were used for statistical and graphical processing of the data.

## Results

**Figure 2:**
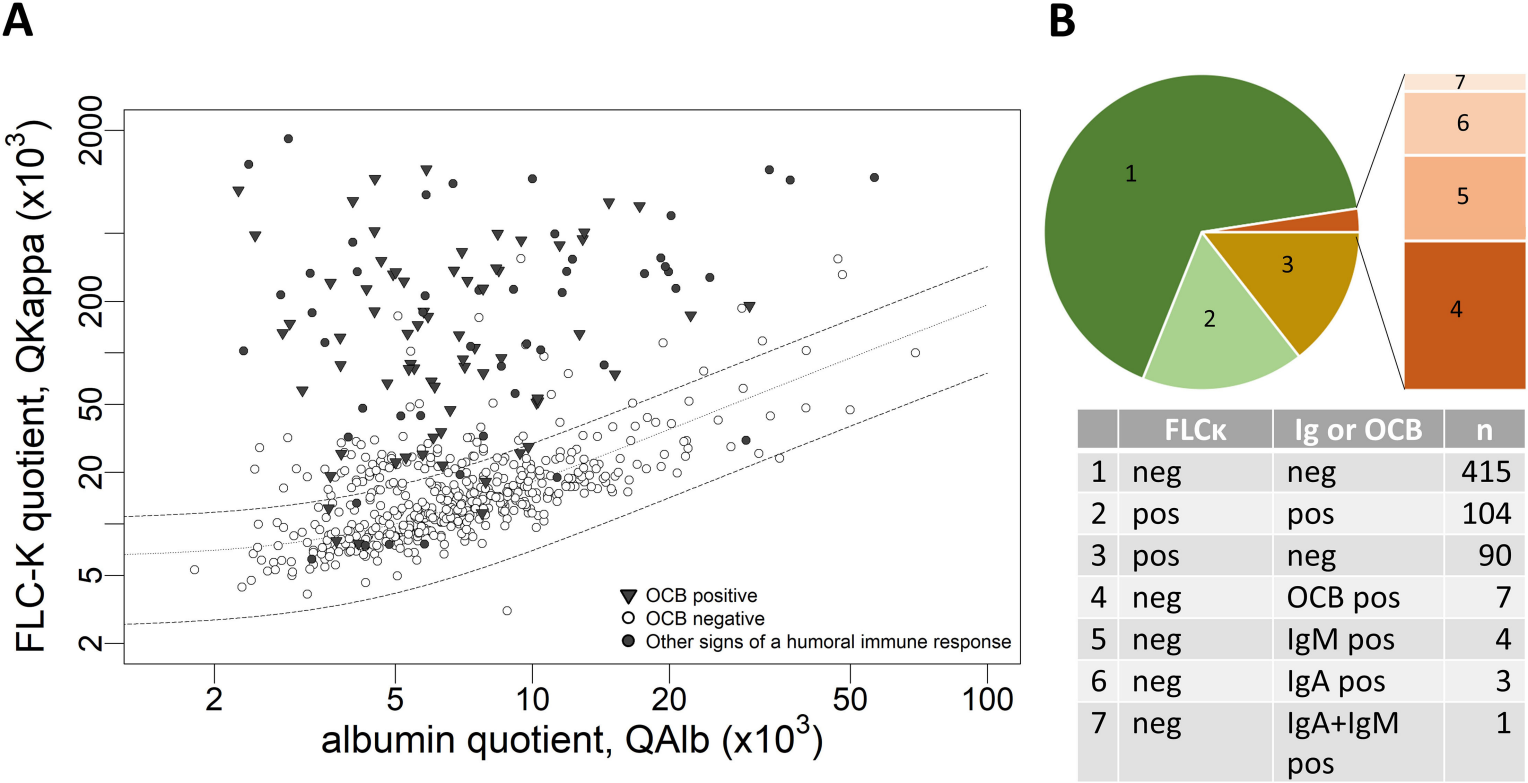
**(A)** Data of the analyzed CSF and serum samples in a double logarithmic FLCκ quotient diagram. Datapoints highlighted in black represent patient samples with an intrathecal humoral immune response (IF IgG, IF IgA, IF IgM > 0% and/or OCB detection in CSF). **(B)** Pie chart showing the proportionate amount of FLCκ IF pos/neg and Ig and/or OCB pos/neg samples. FLCκ free light chains kappa, Q quotient, Alb albumin, CSF cerebrospinal fluid, OCB oligoclonal bands, IF intrathecal fraction.

Laboratory results of the complete cohort are described in **Supplementary Table S4** as well as for the 6 study sites in **Supplementary Table S2**, **Supplementary Table S3**. Thirty-one percent (n=194/624) of the samples had a FLCκ IF > 0% (FLCκ Quotient above the upper limit of the reference range Qlim). Of these, 54% (n=104/194) had additional evidence of humoral intrathecal immune response in the form of CSF-restricted OCB and/or quantitative intrathecal IgG, IgA or IgM synthesis. In 67% of the samples (n=415/624) neither intrathecal FLCκ synthesis nor intrathecal IgG, IgA, IgM synthesis or CSF-restricted OCB were detected, while 15/624 samples were divergent with FLCκ IF ≤ 0% but detection of intrathecal Ig synthesis in quotient diagrams or evidence of CSF restricted OCB (**Figure 2**). Of these 15 samples 7 had CSF-restricted OCB, while 8 samples had an isolated intrathecal IgM and/or IgA synthesis (IgM n=4, IgA n=3 IgM+IgA n=1) (**Table 1**).

**Table 1:** Performance of FLCκ IF (n=624).

	CSF-restricted OCB and/or IF IgG/A/M>0%)	CSF-restricted OCB		No CSF-restricted OCB and IF IgG/A/M<0%	No CSF-restricted OCB		CSF-restricted OCB and/or IF IgG/A/M	OCB
FLCκ IF > 0% FLCκ IF < 0% sensitivity	104	91		90	103	PPV	0.54 (CI 0.47–0.61)	0.47 (CI 0.40–0.54)
	15	7		415	423	NPV	0.97 (CI 0.95–0.98)	0.98 (CI 0.97–1.00)
	0.87 (CI 0.81–0.93)	0.93 (CI 0.88–0.98)	specificity	0.82 (CI 0.79–0.86)	0.80 (CI 0.77–0.84)			
FLCκ Index > 3.61 FLCκ Index < 3.61 Sensitivity	103	90		76	89	PPV	0.58 (CI 0.52-0.63)	0.5 (CI 0.45-0.55)
	16	8		429	437	NPV	0.96 (CI 0.94-0.98)	0.98 (CI 0.97-0.99)
	0.87 (CI 0.79-0.92)	0.92 (CI 0.85-0.96)	specificity	0.85 (CI 0.82-0.88)	0.83 (CI 0.8-0.86)			

CSF cerebrospinal fluid, OCB oligoclonal bands, QIgG/A/M immunoglobulin G/A/M quotient, FLCκ free light chains kappa, IF intrathecal fraction, CI 95% confidence interval.

### Primary outcome parameter

The predefined cut-offs for the primary outcome parameter of ORCAS were a sensitivity of 0.95 to detect - and a NPV of 0.95 to predict the absence of intrathecal Ig synthesis (6). FLCκ IF > 0% was detected in 104 of 119 CSF/serum samples with intrathecal Ig synthesis according to routine diagnostic parameters resulting in a sensitivity of 0.87 (CI 0.81-0.93). This sensitivity is below the predefined cut-off of 0.95. 415 of 430 samples with a FLCκ IF ≤ 0, were also negative for intrathecal Ig synthesis in the routine diagnostic parameters, resulting in a NPV of the FLCκ IF of 0.97 (CI 0.95-0.98).

In an analysis focusing on OCB, the sensitivity of the FLCk IF to detect CSF-restricted OCB was 0.93 (CI 0.88-0.98) and the NPV 0.98 (0.97-1.0) (**Table 1**).

Of note, 61% (n=54/89) of the measurements done with the Optilite platform/The Binding Site FLCκ assay were below the assay specific LLOD.

Due to the integration of the FLCκ Index into the current McDonald criteria for Multiple sclerosis (MS) (9), an additional analysis of this evaluation parameter was performed (**Table 1**). No statistically significant difference was found compared to the use of IF at an Index cut-off of FLCκ I = 3.61 (10) for sensitivity or NPV to detect intrathecal inflammation.

## Discussion

This multicenter real-world study demonstrates a very high NPV confirming the previous single-center study results showing a NPV of 0.99 (5). However, we found that the FLCκ IF has only a moderate sensitivity (0.87) to detect an intrathecal humoral immune response when related to the established routine diagnostic parameters. Thus, the high sensitivity in the monocentric study of 0.98 (discovery cohort) and 0.97 (validation cohort) (5) could not be replicated in this multicenter study. The analysis of the FLCκ Index I = 3.61, which is best suited to detect disease-independent OCB synthesis (10), confirmed these findings.

In 8 samples with false negative FLCκ IF results intrathecal IgA or IgM synthesis was present, while 7 false negative FLCκ IF results occurred in samples with CSF-restricted OCB (**Table 1**). Of note, intrathecal IgM synthesis is particularly susceptible to incorrect nephelometric measurements due to the low concentration in CSF and its high serum gradient (5, 11). Isolated false positive IgM syntheses can also occur in the case of blood contamination (8).

FLCκ CSF data obtained in two laboratories using the Optilite^®^ analyzer and the Freelite^®^ FLC kappa Kit (Binding Site), **Supplementary Table S1**) revealed a proportion of measurements below the LLOD. Other studies using the same platforms and assays have also shown a significant number of FLCκ CSF values below the LLOD up to 64% (12, 13). The clinical relevance of this observation remains to be determined. Previous studies comparing the polyclonal assay (Freelite^®^ FLC kappa Kit, Binding Site) with the monoclonal assay (N Latex FLC kappa Kit, Siemens Healthcare) have shown moderate agreement (10). A meta-analysis concerning the use of the FLCκ Index in MS patients failed to detect any statistically significant difference between the two platforms and assays (14). The primary outcome of this study was explicitly designed to test the comparability of FLCκ performance in daily routine in different laboratories. Further analyses regarding the different methods used are planned in a follow-up investigation on the secondary outcome parameters of this study.

Since a primary endpoint of the present study was sensitivity for the detection of intrathecal Ig synthesis, a large number of patients with high serum FLCκ levels were excluded. When using FLCκ IF as a preselection tool in the general population, there is therefore a risk of further false-negative FLCκ findings.

We conclude that the proposed workflow (5, 6) i.e. using the FLCκ IF as an initial step to identify those CSF/serum sample pairs that do not require additional analysis with Ig quantification and OCB analysis, is not yet recommended due to the risk of overlooking isolated IgA and/or M syntheses or OCB.

The aim of this study was to test the use of FLCκ as a preliminary marker for intrathecal synthesis, regardless of underlying diseases. Therefore, no conclusions can be drawn from the available data regarding its use in MS.

## Summary

This is the first multicenter study to evaluate FLCκ as marker of an intrathecal humoral immune response across different laboratories under routine conditions. As shown in this study, FLCκ can serve as a preliminary marker to identify samples in which an intrathecal humoral immune response is highly unlikely. However, given the limited sensitivity of only 87%, we do not (yet) recommend the exclusive use of FLCκ. For the optimal diagnostic accuracy and comprehensive assessment of an individual patient’s intrathecal humoral immune response, FLCκ analysis should be combined with established measures, including intrathecal Ig synthesis and detection of CSF-specific oligoclonal bands.

## Data Availability

The raw data supporting the conclusions of this article will be made available by the authors, without undue reservation.
