# Supplementary material for: Kappa free light chains in cerebrospinal fluid to detect inflammation - results from a multicentric prospective real-world study
Source: Front Immunol. 2026 Mar 18;17:1806398. doi: 10.3389/fimmu.2026.1806398 (PMC13038534; doi:10.3389/fimmu.2026.1806398)
Supplement: Supplementary file 1 [file DataSheet1.docx]

Supplementary data

Table S1 Methodical data of the 6 study sites in detail

|  | University medicine Greifswald^1^ | Medical school Hannover^2^ | University Hospital Dresden^3^ | Laboratory Limbach, Heidelberg^4^ | SLK-Kliniken Heilbronn^5^ | Medical University Lausitz – Carl Thiem ^6^ |
| --- | --- | --- | --- | --- | --- | --- |
| Platform FLCk measurement | BN ProSpec® (Siemens*) | Atellica® NEPH 630 System (Siemens*) | Atellica® NEPH 630 System (Siemens*) | Optilite® analyser (Binding Site***) | Optilite® analyser (Binding Site***) | BN ProSpec® (Siemens*) |
| FLCk Assay | N Latex FLC kappa Kit* | N Latex FLC kappa Kit* | N Latex FLC kappa Kit* | Freelite® FLC kappa Kit (Binding Site***) | Freelite® FLC kappa Kit (Binding Site***) | N Latex FLC kappa Kit* |
| LLOD FLCk CSF | 0.034 mg/L | 0.034 mg/L | 0.034 mg/L | 0.33 mg/L | 0.29 mg/L | 0.034 mg/L |
| Reference range FLCk serum | 6.7-22.4 mg/L | 6.7-22.4 mg/L | 6.7-22.4 mg/L | 3.30 - 19.4 mg/L | 3.30 – 19.40 mg/L | 6.7-22.4 mg/L |
| OCB method | isoelectric focusing with a semiautomatic agarose electrophoresis system** | isoelectric focusing in polyacrylamide gels followed by silver staining# | isoelectric focusing with a semiautomatic agarose electrophoresis system** | isoelectric focusing with a semiautomatic agarose electrophoresis system** | isoelectric focusing with a semiautomatic agarose electrophoresis system** | isoelectric focusing with a semiautomatic agarose electrophoresis system** |
| Cut-off for OCB positivity | ≥2 bands in CSF | ≥2 bands in CSF | ≥2 bands in CSF | >3 bands in CSF | ≥2 bands in CSF | ≥3 bands in CSF |
| Platform Ig measurement | BN ProSpec® (Siemens*) | Immage® 800, (Beckman Coulter^~^) | cobas®c 501/502 (Roche Diagnostics^+^) | BN ProSpec® (Siemens*) | Optilite® analyser (Binding Site***) | BN ProSpec® (Siemens*) |
| Ethics statement | statement of the ethics committee University medicine Greifswald Vote BB135/22 | statement of the ethics committee Medical School Hannover 1322-2012 | statement of the ethics committee technical university Dresden, Germany BO-EK-480112023 | As this is anonymized laboratory data without clinical information, the Medical care center, Laboratory Dr. Limbach & colleagues GbR, Heidelberg, Germany waived the need for an ethics statement. | statement of the ethics committee regional medical association Baden-Württemberg B-F-2023-044 | statement of the ethics committee medical association Brandenburg |

^1^ Interdisciplinary CSF laboratory, Department of Neurology and Institute of Clinical Chemistry and Laboratory Medicine, University medicine Greifswald, Germany

^2^ Department of Neurology, Medical School Hannover, Hannover, Germany

^3^ Institute of Laboratory Medicine, University Hospital Dresden, Dresden, Germany

^4^ Medical care center, Laboratory Dr. Limbach & colleagues GbR, Heidelberg, Germany

^5^ Institute of Laboratory Medicine, SLK-Kliniken Heilbronn, Heilbronn, Germany

^6^ Institute of Laboratory Medicine, Medical University Lausitz- Carl Thiem, Cottbus, Germany

*Siemens Healthcare Diagnostics Products GmbH, Marburg, Germany

** Hydragel 9 CSF, Sebia Hydrasys 2Scan, Sebia GmbG, Fulda, Germany

*** The Binding Site (Birmingham, UK)

~ Beckman Coulter IMMAGE (CA, USA)

^+^ Roche Diagnostics Corporation (Indianapolis, USA)

^#^ in house method

FLCK free light chains kappa, CSF cerebrospinal fluid, LLOD lower limit of detection, OCB oligoclonal bands, Ig Immunoglobulin

Table S2 Laboratory results of the 6 study sites in detail

|  | University medicine Greifswald^1^ | Medical school Hannover^2^ | University Hospital Dresden^3^ | Laboratory Limbach, Heidelberg^4^ | SLK-Kliniken Heilbronn^5^ | Medical University Lausitz – Carl Thiem ^6^ |
| --- | --- | --- | --- | --- | --- | --- |
| Sample size send | 200 | 100 | 405 | 96 | 52 | 199 |
| Missing data | 4 | 1 | 95 | 3 | 0 | 6 |
| Blood admixture | 3 | 0 | 0 | no data | 0 | 0 |
| Age < 18 y | 0 | 0 | 60 | 0 | 0 | 0 |
| FLCK CSF < LLOD | 0 | 0 | 3 | 57 | 19 | 0 |
| FLCK serum>refrence range (N(%)) | 43 | 28 | 60 | 36 | 19 | 70 |
| Sample size for analysis | 150 | 71 | 187 | 56 | 33 | 127 |
| Age in years (mean (min-max)) | 54 (18-91) | 56 (19-86) | 58 (18-96) | 60 (24-87) | 59 (22-88) | 0 |
| female (n (%)) | 111 (60) | 48 (49) | 138 (58) | 18 (49) | 13 (42) | 99 (53) |
| FLCk serum | 13.75 (11.2;17.4) | 13.80 (10.35; 17.25) | 14.4 (11.5;16.55) | 14.13 (12.01; 16.57) | 14.36 (12.01; 16.24) | 15.1 (12.9;18.4) |
| FLCk CSF | 0.19 (0.12;0.36) | 0.21 (0.13; 0.58) | 0.23 (0.14;0.47) | 0.71 (0.38;2.45) | 0.37 (0.34; 0.66) | 0.21 (0.14; 0.33) |
| FLCk Index | 1.72 (1.39;2.34) | 2.1 (1.6; 4.9) | 2.06 (1.59; 4.72) | 1.92 (0.58; 3.87) | 4.6 (2.8, 6.2) | 2.1 (1.7; 2.9) |
| FLCK IF positivity (n (%)) | 30 (20) | 24 (33.8) | 55 (29.4) | 35 (62.5) | 23 (69.7) | 27 (21.3) |
| OCB pos (n (%)) | 19 (12.7) | 21 (29.6) | 37 (19.8) | 5(9) | 5 (15) | 8 (6.3) |
| WBC (>4/µl) (n (%)) | 1 (1;2) | 1(1;4) | 2(1;4) | no data | 1 (2;5) | 2 (1; 3.5) |
| lactate in mmol/L | 1.9 (1.6;2.1) | 1.71 (1.4; 1.8) | 1.7 (1.5;1.9) | no data | 1.4 (1.3; 1.5) | 1.75 (1.6; 2.1) |
| CSF protein in mg/L | 464 (374;620) | 449.00 (344; 570) | 429 (301;582) | no data | 358 (281; 521) | 433 (353; 572.00) |
| QAlb | 7.1 (5.4;10.6) | 6.5 (4.8, 9.6) | 6.6 (4.6;8.8) | 6.2 (4.5; 8.5) | 5.8 (4.4, 7.9) | 5.9 (4.5; 8.3) |
| QIgG | 3.15 (2.3;4.6) | 3.7 (2.7; 5.5) | 3.2 (2.2;4.5) | 3 (2.1; 4.4) | 2.9 (2.2;5) | 3.1 (2.3;4.7) |
| QIgA | 1.8 (1.3;3) | 1.84 (1.3; 2.8) | 1.6 (1.1;2.7) | 1.5 (1; 2.5) | 1.5 (1; 2.4) | 1.5 (1.1; 2.2) |
| QIgM | 0.5 (0.3; 1.05) | 0.4 (0.3; 0.7) | 0.38 (0.21; 0.74) | 0.25 (0.08; 0.63) | 0.4 (0.23; 0.57) | 0.3 (0.2; 0.6) |
| IF IgG > 0% (n (%)) | 3 (2) | 8 (11.3) | 13 (7) | 5 (5.8) | 5 (15.2) | 13 (7) |
| IF IgA > 0% (n (%)) | 4 (2.7) | 2 (2.8) | 5 (2.7) | 2 (3.6) | 1 (3) | 5 (2.7) |
| IF IgM > 0% (n (%)) | 5 (3.3) | 12 (16.9) | 15 (8) | 1 (1.8) | 1 (3) | 8 (6.3) |

Continuous data are expressed as median (first; third quartiles) unless otherwise specified; nominal data are given as percentages

^1^ Interdisciplinary CSF laboratory, Departement of Neurology and Institute of Clinical Chemistry and Laboratory Medicine, University medicine Greifswald, Germany

^2^ Department of Neurology, Medical School Hannover, Hannover, Germany

^3^ Institute of Laboratory Medicine, University Hospital Dresden, Dresden, Germany

^4^ Medical care center, Laboratory Dr. Limbach & colleagues GbR, Heidelberg, Germany

^5^ Institute of Laboratory Medicine, SLK-Kliniken Heilbronn, Heilbronn, Germany

^6^ Institute of Laboratory Medicine, Medical University Lausitz- Carl Thiem, Cottbus, Germany

FLCK free light chains kappa, CSF cerebrospinal fluid, LLOD lower limit of detection, OCB oligoclonal bands, Ig Immunoglobulin, IF intrathecal fraction, WBC white blood cell count

Table S3 Results of the contingency tables from the 6 study sites

| Study site | FLCk IF pos/OCB+Ig pos | FLCk IF pos/OCB+Ig neg | | FLCk IF neg/OCB+Ig pos | FLCk IF neg/OCB+ Ig neg | Sens | NPV | OR (95% CI) | N (total) |
| --- | --- | --- | --- | --- | --- | --- | --- | --- | --- |
| 1 | 18 | 12 | | 2 | 118 | 0.9 | 0.98 | 88.5 (18.28-428.36) | 150 |
| 2 | 8 | 47 | | 1 | 131 | 0.89 | 0.99 | 22.298 (2.72-183.07) | 187 |
| 3 | 11 | 16 | | 3 | 97 | 0.79 | 0.97 | 22.23 (5,58-88.52) | 127 |
| 4 | 22 | 2 | | 3 | 44 | 0.88 | 0.94 | 161.33 (25.09-1037.35) | 71 |
| 5 | 6 | 17 | | 1 | 9 | 0.86 | 0.9 | 3.18 (0.33-30.62) | 33 |
| 6 | 7 | 28 | | 0 | 21 | 1 | 1 | RR 0.8 (0.68-0.94) | 56 |
| pooled OR (Mantel-Haenszel) | |  | Estimate 33.16 95% CI 14.36-76.6 p value <.001 | | | | | | |
| Mantel-Haenszel ꭓ² | |  | Estimate 149.85, p-value <.001 | | | | | | |

^1^ Interdisciplinary CSF laboratory, Department of Neurology and Institute of Clinical Chemistry and Laboratory Medicine, University medicine Greifswald, Germany

^2^ Institute of Laboratory Medicine, University Hospital Dresden, Dresden, Germany

^3^ Institute of Laboratory Medicine, Medical University Lausitz- Carl Thiem, Cottbus, Germany

^4^ Department of Neurology, Medical School Hannover, Hannover, Germany

^5^ Institute of Laboratory Medicine, SLK-Kliniken Heilbronn, Heilbronn, Germany

^6^ Medical care center, Laboratory Dr. Limbach & colleagues GbR, Heidelberg, Germany

FLCK free light chains kappa, OCB oligoclonal bands, Ig Immunoglobulin, IF intrathecal fraction, Sens Sensitivity, NPV negative predictive value, OR odds ratio

Table S4 Baseline characteristics and cerebrospinal fluid results of all patient samples

|  |  |
| --- | --- |
| Sample size for analysis | n=624 |
| Age in years (median (min-max)) | 48 (32-60) |
| Female (n (%)) | 372 (59) |
| FLCκ serum | 14.2 (12.01;16.35) |
| FLCκ CSF | 2.39 (0.35;2.58) |
| FLCκ IF >0% (n (%)) | 194 (31) |
| OCB pos* (n (%)) | 98 (16) |
| Lactate in mmol/L | 1.88 (1.6;2.1) |
| CSF protein in mg/L | 439 (281;524) |
| QAlb | 7.41 (4.4;8.4) |
| QIgG | 4.5 (2.2; 4.6) |
| QIgA | 1.5 (1;2.5) |
| QIgM | 0.7 (0.2;0.6) |
| IF IgG > 0% (n (%)) | 39 (6.25) |
| IF IgA > 0% (n (%)) | 19 (3) |
| IF IgM > 0% (n (%)) | 42 (6.7) |

Continuous data are expressed as median (first; third quartiles) unless otherwise specified; nominal data are given as percentages.

CSF cerebrospinal fluid, OCB oligoclonal bands, QIgG/A/M immunoglobulin G/A/M quotient, QAlb albumin quotient, FLCκ free light chains kappa, IF intrathecal fraction

* presence of OCB solely in CSF according to local standards
